# Malignant struma ovarii with thyroid-type papillary and poorly differentiated carcinoma: a case report 

**DOI:** 10.1186/s13256-022-03590-6

**Published:** 2022-09-30

**Authors:** Nao Terayama, Satoe Fujiwara, Shoko Ueda, Takashi Yamada, Masahide Ohmichi

**Affiliations:** 1 Department of Obstetrics and Gynecology, Osaka Medical and Pharmaceutical University, 2-7 Daigakumachi, Takatsuki, Osaka 569-8686 Japan; 2 Department of Pathology, Osaka Medical and Pharmaceutical University, Osaka, Japan

**Keywords:** Malignant struma ovarii, Papillary thyroid carcinoma, Poorly differentiated thyroid carcinoma, Adjuvant therapy

## Abstract

**Background:**

Malignant struma ovarii is a very rare type of gynecologic cancer. Although its most common histological subtype is a pure type of papillary thyroid carcinoma containing two components, papillary carcinoma and poorly differentiated carcinoma, malignant struma ovarii is still extremely rare. As a result, the optimal treatment for this type of tumor remains uncertain due to its rarity.

**Case presentation:**

A 62-year-old Japanese female presented with a pelvic tumor and clinical diagnosis of malignant tumor of the ovary. She underwent complete debulking surgery, total abdominal hysterectomy, bilateral salpingo-oophorectomy, and omentectomy. The histology of the ovarian tumor revealed malignant struma ovarii with thyroid-type papillary projections and poorly differentiated carcinoma. Because of the complete resection and the absence of distant metastasis, the patient did not receive any adjuvant therapy. At 24 months after surgery, she was free of disease.

**Conclusion:**

This is a rare case report of malignant struma ovarii, without recurrence, in which the component was papillary thyroid carcinoma mixed with poorly differentiated carcinoma. Foregoing adjuvant therapy might be one option for malignant struma ovarii in cases with complete resection and no distant metastasis. In addition, we should consider that long-term follow-up is needed for malignant struma ovarii.

## Background

Struma ovarii (SO) is a very rare disease, defined as a single germ cell tumor consisting ≥ 50% thyroid tissue, thus making it distinguishable from mature teratoma [1, 2]. Malignant struma ovarii (MSO) is an even rarer tumor, accounting for approximately 5–10% of SO cases that are histologically identified as differentiated thyroid carcinoma [3]. To date, only about 200 cases have been reported in literature [4]. The most common histological type of MSO is papillary carcinoma, which accounts for about 50% of MSO cases [5, 6]. On the other hand, a pure type of poorly differentiated carcinoma accounts for about 3%, and mixed follicular and papillary carcinoma accounts for about 3% as well [3, 7]. Due to this tumor’s rarity, the optimal treatment of MSO remains uncertain.

We present herein a case report of malignant struma ovarii without recurrence in which the component was papillary carcinoma mixed with poorly differentiated carcinoma.

## Case presentation

**Fig. 1:**
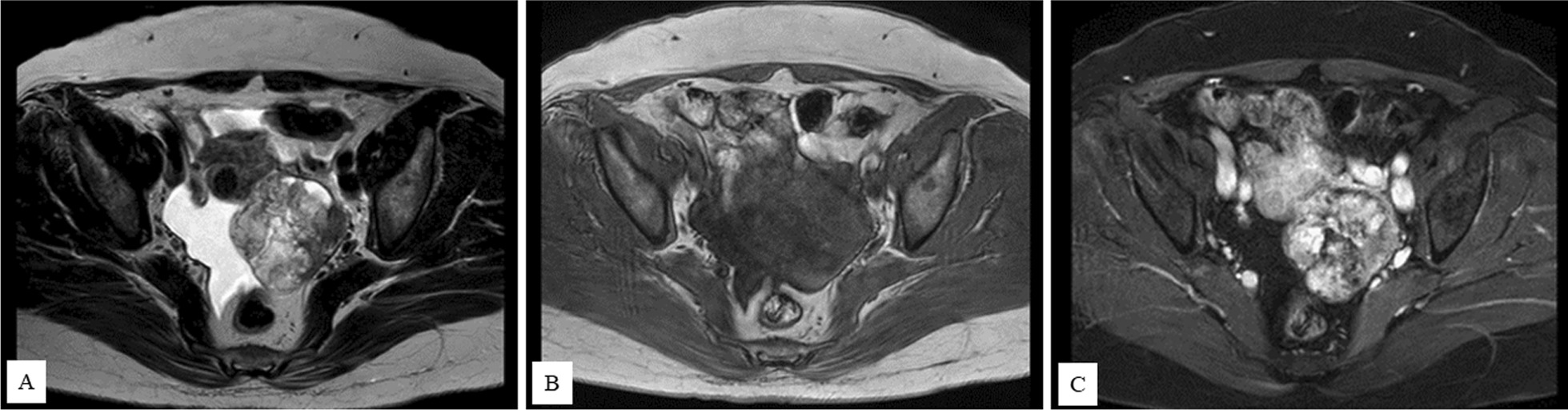
Magnetic resonance imaging findings. **A** T2-weighted image revealing a multilocular cystic tumor with solid components and a diameter of 6 cm in the left ovary. There was a small volume of ascites. The tumor showed heterogeneity in T2-weighted imaging. **B** In T1-weighted image, the tumor showed mostly low signal but some high signal. **C** In T1 fat-suppressed and contrast imaging, the tumor showed heterogeneous staining in the low-signal part in T1-weighted image

A 62-year-old Japanese female (gravida 4, para 3) complained of 1-month history of abdominal distension. She had no medical history. Physical examination revealed a pelvic mass touched on the dorsal side of the uterus. Transvaginal ultrasound revealed a 6  × 5 cm^2^ cyst with solid parts on the left side of the uterus. Subsequent magnetic resonance imaging (MRI) revealed a multilocular cyst tumor, with solid components and a diameter of 6 cm, in the left ovary, and small-volume ascites. The tumor showed heterogeneity on the T2-weighted image and mostly low signal, but some high signal, which was not fat suppressed in T1-weighted images after fat suppression (Fig. 1). Computed tomography (CT) showed no distant metastasis and no obvious abnormal accumulation in the thyroid gland. The patient’s preoperative serum level of cancer antigen 125 (CA125) was elevated to 84.9 U/ml (normal range <35.0), whereas the carcinoembryonic antigen (CEA), cancer antigen 19-9 (CA19-9), and squamous cell carcinoma-related antigen (SCC) were within their respective normal ranges.

Total abdominal hysterectomy, bilateral adnexectomy, and omentectomy were performed without any residual tumor because the intraoperative rapid histological diagnosis of the left ovarian tumor was suspicious for low-grade malignancy. The postoperative course, as well, was uneventful.

**Fig. 2:**
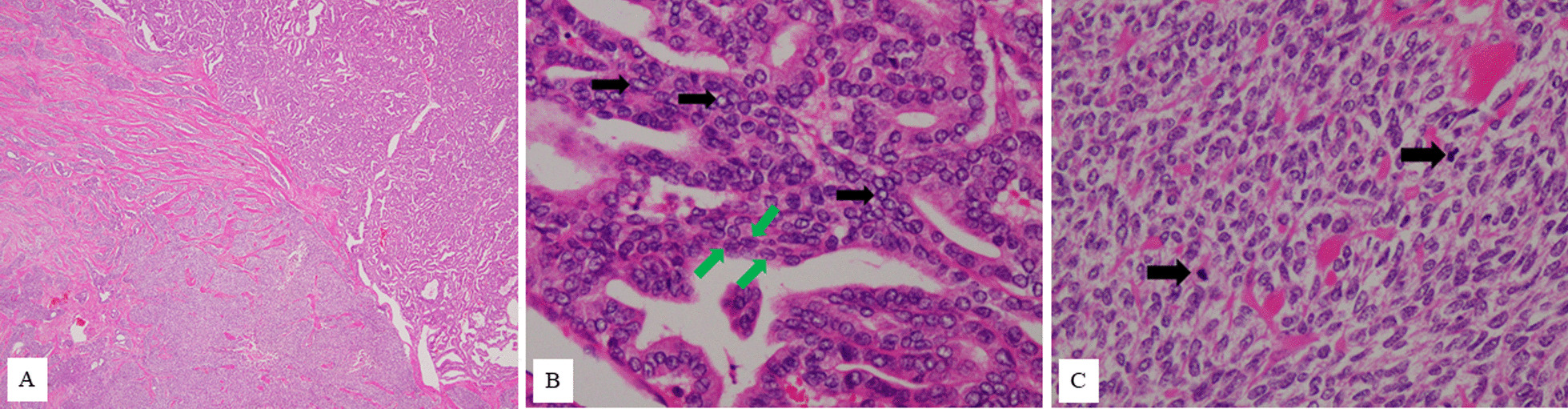
Histological image of the ovary. **A** Papillary thyroid carcinoma on the upper right corner of the screen shows papillary growth. The lower left of the screen shows poorly differentiated carcinoma. Chordal arrangement, insular arrangement, and solid patterns are seen (HE: ×40). **B** In the part of papillary thyroid carcinoma, high-resolution image depicting the cells comprising the papillary architecture of the tumor. The nuclei of the tumor cells are large, and the nuclear fission image is inconspicuous. Ground-glass nuclei (black arrows) and coffee-bean like nuclear grooves (green arrows) are seen. (HE: ×400) **C** In the part of poorly differentiated carcinoma, solid patterns composed of unified cells with increased mitotic activity (arrows) (HE:×400)

Upon gross examination of the surgically resected specimen, the left ovary was whitish with tough elasticity, and the split surface was composed of polycystic formations with a solid area. Histologically, there were two patterns of histological subtypes: papillary carcinoma and poorly differentiated carcinoma (Fig. 2A). About 70% of the tumor was composed of follicular epithelium of the thyroid gland growing in a papillary fashion, while about 30% of the tumor had poorly differentiated carcinoma. In the area of the papillary carcinoma, the nuclei of the tumor cells were like “ground glass” with coffee bean-like grooves (Fig. 2B). In the other area, full, nodular, and insular structures were observed, and nuclear fission images were confirmed (Fig. 2C). Based on these findings, we made a diagnosis of malignant struma ovarii, as shown by the histological type of papillary carcinoma mixed with poorly differentiated carcinoma. No teratoma component was identified. The immunohistochemical analysis showed that both components were positive for thyroglobulin; the Ki-67 (MIB-1) index was 5% in the papillary carcinoma and 35% in the poorly differentiated carcinoma.

The final diagnosis was MSO classified as stage IA (pT1aNxM0), according to the International Federation Gynecology and Obstetrics (FIGO) 2014 classification, with histological type of papillary carcinoma mixed with poorly differentiated carcinoma. The patient was subsequently referred to the Department of Otorhinolaryngology to confirm whether the ovarian tumor originated from a primary thyroid tumor. Adenomatous goiter was suspected upon ultrasound, and the patient underwent examination of the thyroid gland by fine-needle aspiration cytology. The results were negative for thyroid cancer. We considered postoperative adjuvant therapy, thyroidectomy, and radioactive iodine ablation (RIA). Since there were no distant metastases and no malignant findings in the thyroid gland, we decided to follow up the patient without adjuvant therapy.

The patient is scheduled for follow-up with postoperative serial measurements of thyroglobulin and serum CA125 every 2–3 months, contrast-enhanced computed tomography twice a year, and thyroid ultrasonography once a year. The patient was free of disease at 24-month follow-up consultation without any side effects.

## Discussion

Malignant struma ovarii (MSO) is a rare tumor. The survival outcome of MSO is limited and, therefore, no management strategy has been established.

The most common histological type of MSO is papillary carcinoma, which accounts for about 50% [2, 3]. The other histological types are follicular degeneration of papillary carcinoma (20–30%), follicular carcinoma (20%), mixed follicular and papillary carcinoma (about 3%), poorly differentiated carcinoma (about 3%), anaplastic carcinoma (about 1%), and medullary carcinoma (about 1%) [2, 3]. If the tumor is completely composed of thyroid carcinoma, it is named “pure” MSO; if the tumor has teratoma components, it is named “impure” MSO. Pure types account for 30% of MSO, while impure types account for 70% [2].

The diagnostic criteria for MSO have been in accordance with those for typical thyroid-type cancers. In follicular neoplasms of the thyroid gland, invasion of the tumor into the capsule is also considered a sign of malignancy. Robboy *et al*. reported that, since the ovary does not have a capsule, extension through the irregular fibrous thickening of the outer cortex, which may occur above an ovarian tumor, represents invasion of a true capsule [1]. Papillary carcinoma, which accounts for about 50% of MSO, can be diagnosed by the presence of a papillary structure and a recognized “ground glass” nuclei. However, in the absence of true papillary structures, the presence of glassy nuclei alone is insufficient to diagnose papillary carcinoma of struma ovarii [1]. It is not common for vascular invasion to be observed. The presence of ovarian serous invasion is an important clue to the diagnosis [1, 8]. Poorly differentiated thyroid carcinoma is a rare type, even in primary thyroid cancer, and extremely rare in struma ovarii, which account for only about 3% of MSO [2, 3, 8]. For many years, the diagnostic criteria for poorly differentiated thyroid cancer have been controversial owing to their rarity [9]. The hallmarks of poorly differentiated carcinomas are a uniform population of small follicular cells arranged in a solid, trabecular (or insular) pattern. It is also commonly characterized by significant mitotic activity (more than 3 in 10 high-power fields), necrosis or convoluted nuclei, and the absence of typical nuclear characteristics of papillary carcinoma [9]. In our case, the nuclei of the tumor cells were “ground glass” with coffee bean-like grooves in the area of the papillary carcinoma. Moreover, in the poorly differentiated area, full, nodular, and insular structures were observed, and nuclear fission images were confirmed. Based on these findings, we diagnosed the patient as having malignant struma ovarii, as evidenced by the histological type of papillary carcinoma mixed with poorly differentiated carcinoma. No teratoma component was identified, so this case was a pure type.

No standard therapy has been established for MSO throughout the published literature. As surgical treatment for MSO, total abdominal hysterectomy, bilateral adnexectomy, and omentectomy are considered optimal [10]. Some reports have revealed that more aggressive treatment did not reduce the rate of tumor recurrence, so it was suggested that unilateral salpingo-oophorectomy/unilateral oophorectomy be performed to preserve the patient’s fertility in this case [1, 4, 11]. In the 68 patients analyzed by Goffredo *et al*., lymph nodes were often not examined (73%), and even with the lymph nodes that were examined, none of them were confirmed to have any metastasis [4]. Therefore, after the final histological diagnosis, we decided that additional lymph node dissection was not necessary. Our patient received total abdominal hysterectomy, bilateral adnexectomy, and omentectomy, which is consistent with published literature [1, 4].

In addition, no guidelines for postoperative adjuvant therapy are defined either. Thyroidectomy and radioactive iodine ablation (RIA) have been discussed [12, 13], and some authors have promoted routine RIA as a means to reduce the recurrence rate after surgery [11, 14]. Yassa *et al*. suggested that patients who have extra-ovarian disease or aggressive histological features should consider a thyroidectomy and eventually undergo RIA [14]. On the other hand, some authors have suggested that RIA should be performed in patients with distant metastasis because their prognosis in poor [4, 13]. Although there are no histological or clinical features that can reliably predict which tumors are biologically malignant, dense fibrous adhesions, peritoneal fluid, and large stroma (especially >12 cm) are suggestive of a tumor that has spread by the time of surgery or is likely to recur [15]. It remains controversial whether to perform RIA in patients with MSO at stage IA [3]. In our case, we did not perform adjuvant therapy, including thyroidectomy and RIA, because there was no distant metastasis, no malignant cells in the ascites, and no dense fibrous adhesions; moreover, the tumor size was small (<12 cm).

Postoperative monitoring of MSO is even more important in order to rule out the possibility of metastasis [16]. Therefore, it is important to evaluate serum thyroglobulin in a long-term follow-up in cases of thyroid cancer [3, 7, 10]. Li *et al*. recommended that all patients with MSO should have serum thyroglobulin assessments every 6–12 months [3]. The most common site of metastasis was the peritoneum, followed by the liver, lungs, and bones [3, 17]. There is a report that recommended regular follow-up by contrast-enhanced CT of the chest, abdomen, and pelvis twice or three times a year [16].

Robboy *et al*. reported that the overall survival (OS) rate for all patients at all stages was 89% at 10 years and 84% at 25 years [15]. Similarly, a study including 68 cases of MSO found OS rates at 5, 10, and 20 years of 97%, 94%, and 85%, respectively [4]. Based on these findings, they suggested routine long-term follow-up [4, 15]. On the other hand, there have been no reports on prognosis according to stage. The median time to recurrence has been reported to be 4 years [14], and it is also recommended that monitoring of MSO should take place for at least 20 years because the median duration of recurrence is 14 years, and cases of late recurrence have been reported [3]. This means that long-term follow-up care is required for MSO.

## Conclusion

This is a rare case report of MSO with papillary carcinoma mixed with poorly differentiated carcinoma. In this case, a good clinical course was obtained by complete resection without adjuvant therapy. In addition, we have to consider that long term follow-up is needed for MSO.

## Data Availability

The data used or analyzed are all included in this published article.

## Abbreviations

SO: Struma ovarii
MSO: Malignant struma ovarii
CA125: Cancer antigen 125
FIGO: International Federation of Gynecology and Obstetrics
